# Skin Color in Apple Fruit (*Malus × domestica*): Genetic and Epigenetic Insights

**DOI:** 10.3390/epigenomes4030013

**Published:** 2020-07-13

**Authors:** Wuqian Wang, Jean-Marc Celton, Gerhard Buck-Sorlin, Sandrine Balzergue, Etienne Bucher, François Laurens

**Affiliations:** 1 IRHS (Institut de Recherche en Horticulture et Semences), UMR 1345, INRAE, Agrocampus-Ouest, Université d’Angers, SFR 4207 QuaSaV, F-49071 Beaucouzé, France; wuqian.wang@inrae.fr (W.W.); jean-marc.celton@inrae.fr (J.-M.C.); gerhard.buck-sorlin@agrocampus-ouest.fr (G.B.-S.); sandrine.balzergue@inrae.fr (S.B.); 2 Plant Breeding and Genetic Resources, Agroscope, 1260 Nyon, Switzerland; etienne.bucher@agroscope.admin.ch

**Keywords:** epigenetics, genetics, skin color, apple, transposable element, transcriptome factor

## Abstract

Apple skin color is an important trait for organoleptic quality. In fact, it has a major influence on consumer choice. Skin color is, thus, one of the most important criteria taken into account by breeders. For apples, most novel varieties are so-called “mutants” or “sports” that have been identified in clonal populations. Indeed, many “sports” exist that show distinct phenotypic differences compared to the varieties from which they originated. These differences affect a limited number of traits of economic importance, including skin color. Until recently, the detailed genetic or epigenetic changes resulting in heritable phenotypic changes in sports was largely unknown. Recent technological advances and the availability of several high-quality apple genomes now provide the bases to understand the exact nature of the underlying molecular changes that are responsible for the observed phenotypic changes observed in sports. The present review investigates the molecular nature of sports affected in apple skin color giving arguments in favor of the genetic or epigenetic explanatory models.

## 1. Introduction

Apple skin color is one of the most important factors determining the acceptance and economic value of apples. The red coloration of the skin of an apple fruit is mainly attributed to anthocyanin pigmentation. Therefore, anthocyanin is key to improving our understanding of the genes and mechanisms involved in this trait.

In Europe, distinctness, uniformity, and stability (DUS) testing of fruit species is long and expensive compared to other crop sectors [1]. This process aims to determine whether a new variety is different from an existing variety within the same species (“distinctness”). Furthermore, it is used to determine whether distinctive traits are expressed uniformly (“uniformity”), and whether subsequent processes will change the phenotype of the generation (“stability”). The DUS test exists to allow new varieties to enter the market legally and allow plant breeders to protect their rights. Apple varieties appear to come about as clearly distinctive mutants, but certain apple mutants, for example derived from the “Gala” variety, may only show subtle phenotypic changes and are difficult to distinguish from each other, thus requiring long and expensive tests. To better distinguish these varieties, it is necessary to decipher the genetic or epigenetic origin of the skin color mutations, then to develop molecular genetic or epigenetic markers to help institutions such as the Community Plant Variety Office (CPVO) to accelerate testing of fruit species.

Recently, numerous studies have investigated the roles of genetics and epigenetics in apple skin color development. The aim of this review is to bring together the findings of these articles and summarize the genetic and epigenetic regulation of apple skin color. This review gives arguments in favor of the genetic and epigenetic explanatory models and gives an overview of the research frontier.

## 2. Anthocyanin Biosynthesis and Pigment Composition

Apple skin color is determined by the contents of anthocyanins, carotenoids, and chlorophyll, as well as their distribution over the skin surface. Anthocyanin content plays an important role in the degree of coloring of red apples. The content and distribution patterns of anthocyanins in the skin are responsible for the different color phenotypes. With the same anthocyanin content, a higher chlorophyll content will make the skin color dark red, whereas a lower content will cause the skin color to be bright red [2,3].

Anthocyanins are water-soluble flavonoids occurring naturally in plants and are widely distributed. Furthermore, anthocyanins are important in plants as secondary metabolites. So far, twenty-two major types of anthocyanins have been described in more than 250 species [4]. At present, five anthocyanin components have been studied extensively in the skin of apples: cyanidin-3-arabinoside, cyanidin-3-rutinoside, cyanidin 3-galactosides, cyanidin-3-xylosides, and cyanidin-3-glucosides [5]. Among these anthocyanins, cyanidin-3-galactoside was found to be the most abundant in apple skin, accounting for about 80% of the total anthocyanin content [4].

Anthocyanins play numerous roles in plants, most importantly in the reproductive organs. For example, anthocyanins can make flowers appear to be brightly colored to facilitate plant pollination [6]. In addition, anthocyanins can protect plants from low temperatures and other abiotic and biotic stresses [7]. Indeed, when plants are in a stressful environment, the accumulation of anthocyanins can have a protective role [8]. A well-known example is the reddening of the leaves of deciduous trees in autumn, induced by lower temperatures, to protect the plant from freezing damage [9]. In addition, studies have shown that anthocyanins can play an antioxidant role in plants; however, the proportion of anthocyanins with an antioxidant function in apples is only about 1–20% [10]. Anthocyanins are also an important indicator for evaluating fruit maturity. Generally, the content of anthocyanins will be relatively high when the fruit is mature.

**Figure 1:**
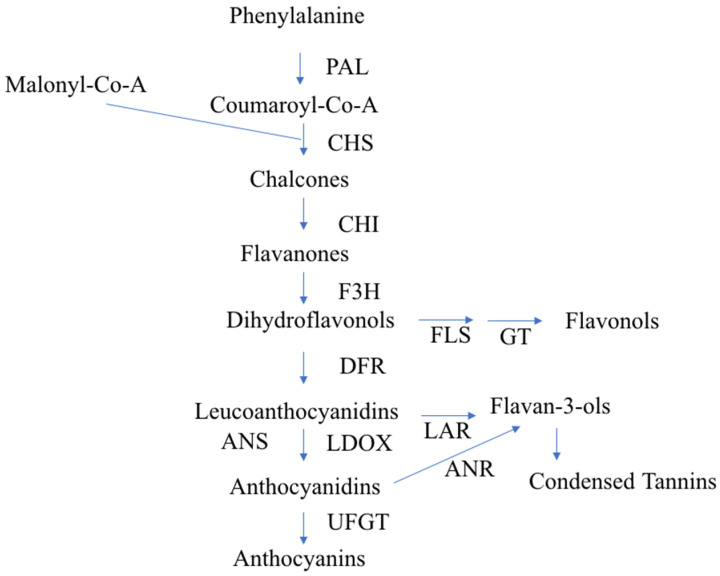
Diagrammatic representation of the flavonoid biosynthetic pathway in apples, according to Takos et al. [14]. PAL, Phe ammonia lyase; CHS, Chalcone synthase; CHI, chalcone isomerase; F3H, flavanone-3 b-hydroxylase; DFR, dihydroflavonol-4-reductase; LDOX, leucoanthocyanidin dioxygenase. UFGT, UDP-glycose: flavonoid-3-*O*-glycosyltransferase; FLS, flavonol synthase; GT, glycosyl transferase; LAR, leucoanthocyanidin reductase; ANR, anthocyanidin reductase.

To study fruit color, we first need to inspect the flavonoid biosynthesis pathway. This pathway has already been studied in detail—it starts with the biosynthesis of phenylalanine with the help of different enzymes and transcription factors (TFs) [11] (Figure 1).

The biosynthesis of anthocyanin pigments is influenced by two categories of genes. To the first category belong structural genes encoding enzymes, such as chalcone synthase (CHS), chalcone isomerase (CHI), flavanone-3-hydroxylase (F3H), dihydroflavonol 4-reductase (DFR), leucoanthocyanidin dioxygenase (LDOX), and UDP-glucose: flavonoid-3-*O*-glycosyltransferase (UFGT). The second category comprises genes coding for regulatory proteins, which include a number of TFs that influence the intensity and pattern of anthocyanin accumulation. At least three protein families, namely MYB, bHLH, and WD40, are involved in the regulation of anthocyanin synthesis, but the specific classes and the genes involved in the biosynthesis vary depending on the species [12,13].

Anthocyanins can accumulate in leaves, roots, and stocks [15], while regulatory proteins which involve in the flavonoid biosynthesis pathway are localized in the cytosol. After biosynthesis, flavonoids are transported to vacuoles or cell walls [16]. Anthocyanins are upregulated by maturity and the formation of flavonoids, especially anthocyanin and chlorogenic acid in apple skin, which influence growth regulators and fruit maturity [17]. The signals or metabolite biosynthesis which influence the maturity of fruits also influence anthocyanin accumulation. For example, plant hormones (abscisic acid, ethylene, jasmonates, auxin, gibberellin) have a crucial role in the regulation of fruit development and ripening. They interact with MYB–bHLH–WD40 complexes at either the transcriptional or the post-transcriptional level to control the anthocyanin composite [18]. Espley et al. [15] reported that the increased flux in the anthocyanin pathway was also associated with increases in metabolite concentrations of other polyphenols, including up to a 10-fold increase in quercetin-3-galactoside. Anthocyanins in the vacuole are degraded by peroxidases. The possible involvement of vacuolar peroxidases in anthocyanin degradation may be a component of the adaptation of plants to changing environmental conditions, such as decreased light intensities, but may also be a component of the plant developmental program [19].

Rootstocks also link with apple skin color. From the 20th century, much research has been conducted on the subject. In a study using seven rootstocks, Rogers [20] observed the best color on fruit from “Bramley” trees grafted on “M9”. Tukey and Brase [21] and Blair [22] observed that red-colored cultivars developed higher color on “M”. Hewetson [23] and Upshall [24] found that the increase in red fruit color intensity found on some Malling rootstocks was due to earlier maturity date [25]. The amount of total phenolic compounds and flavonoids increased, which may be due to the influence of different rootstocks and the incompatibility between the rootstock and the scion or grafting wound. In one study, “Bud 9” rootstock generally had a negative effect on the amount of anthocyanin, while M9 increased the amount of anthocyanin [26]. Grafting of “Bekran” on “Bekran” rootstock and “Red Delicious” cultivar on “Bekran” decreased the anthocyanin in scion leaves, but when “Bastam” cultivar was grafted on this rootstock it increased the amount of anthocyanin [26].

## 3. Genetic Determinants of Apple Skin Color

### 3.1. MYB Transcription Factors Regulate Apple Skin Color

The genetic mechanisms determining skin color in apple have been investigated in a number of studies [14,27,28]. MYB TFs have been reported to play an important role in plant secondary metabolism, especially the phenylalanine metabolism pathway. In particular, the well-studied *MdMYB1*, *MdMYBA*, and *MdMYB10* genes code for TFs that were found to control the color of apple skin and flesh [14,27,28].

*MdMYB1* (Md: *Malus × domestica*) is a TF isolated and identified from apples that regulates anthocyanin biosynthesis in apple skins. The transcriptional abundance of this gene was strongly regulated by light and positively correlated with anthocyanin accumulation [14]. Another study confirmed that light can make the *MdMYB1* protein more stable, and therefore can enhance the anthocyanin synthesis of apple skin [29]. A further finding was that the promoter region of *MdMYB1* is rich in polymorphisms [14]. Based on these polymorphisms, a set of dCAPS markers was designed based on *MdMYB1-1*, which had a dominant effect on fruit skin color [14]. Apples that have *MdMYB1-1* will exhibit a red-skinned phenotype, whereas those that do not have the *MdMYB1-1* allele will have yellow or green skin [14]. However, this *MdMYB1* dCAPS marker was unable to identify alleles of the *MdMYB1* gene in “Fuji” apples [30].

In addition to *MdMYB1*, *MdMYB10* and *MdMYBA* were isolated and found to be closely associated with the redness of apple skin and flesh [27,28]. Low temperature and UV-B (280 ~ 320nm) induced *MdMYBA* in different tissues. There were significant differences in expression levels between differently colored varieties [27]. Confirming its direct role in anthocyanin biosynthesis, it was shown that *MdMYB10* overexpression could significantly enhance anthocyanin accumulation in transgenic apple seedlings [28].

**Figure 2:**
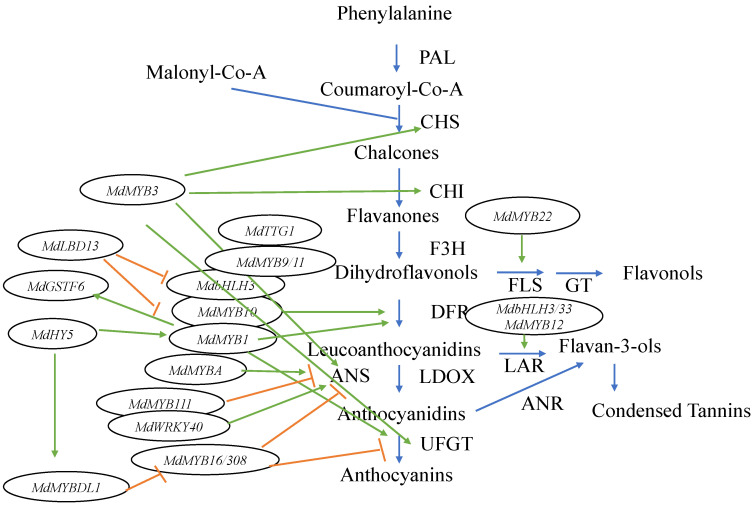
Diagrammatic representation of the anthocyanin biosynthetic pathway and the related TFs in apples. Green arrows indicate TFs activating enzymes in the pathway, while orange barred lines signify a TF inhibiting enzyme expression in the pathway.

In recent research, the anthocyanin biosynthesis process has been proven to be more complex, with more genes, TFs, and interactions having been discovered. A number of other MYB TFs were found in the apple genome (Figure 2). Among them, *MdMYB3* is involved in the transcriptional activation of several flavonoid biosynthesis pathway genes [31]. In addition, it not only regulates anthocyanin accumulation in the apple skin, but also participates in the regulation of flower development [31]. Recently, another MYB TF, *MdMYB16*, was described, which could form homodimers and directly inhibit anthocyanin synthesis through its C-terminal EAR (ethylene responsive element binding factor-associated amphiphilic repression) repressor [32]. *MdMYB16* was shown to interact with a bHLH family TF *MdbHLH33* to weaken inhibition of anthocyanin synthesis [32]. More recently, a light-responsive MYB-like gene called *MdMYBDL1* was found [33]. It functions downstream of *MdHY5*, which coordinates light signal transduction and regulates the expression of flower color [33]. *MdHY5* activates the expression of *MdMYBDL1*, while *MdMYBDL1* inhibited the transcription of *MdMYB16* and its homolog *MdMYB308*, which are inhibitors of anthocyanin biosynthesis in apples. This means that *MdHY5* is able to promote anthocyanin biosynthesis in apples (Figure 2) [32]. These results indicate that *MdHY5* enhances apple anthocyanins synthesis by acting on different types of MYB TFs [33]. In addition, *MdMYB9* and *MdMYB11* were found to bind to the promoters of structural genes, such as a bHLH TF *MdbHLH3* and WD repeat protein TTG1 to regulate anthocyanin and PA accumulation [34]. Moreover, two putative flavonoid-related genes, *MdMYB12* (proanthocyanidin-specific TF) and *MdMYB22* (flavonol-specific TF), were found to directly facilitate the expression of leucoanthocyanidin reductase (LAR) and flavonol synthase (FLS), respectively [35]. *MdMYB12* is thought to interact with *bHLH3* and *bHLH33*, which are regulators of anthocyanin synthesis in apples [28], and to facilitate the expression of LAR and promote proanthocyanidin synthesis. Furthermore, *MdMYB22* activates flavonol pathways by directly combining with the flavonol synthase (FLS) promoter [35]. Among the MYB TFs, *MdMYB111* was found to bind the MYB recognition element (MRE) of the *MdANS* (anthocyanidin synthase) promoter and to potentially inhibit anthocyanin biosynthesis [36]. *MdWRKY40* was found to form homodimers and to bind to the W box of the *MdANS* promoter. In addition, it was shown to reduce the inhibitory effect of *MdMYB111* on anthocyanin biosynthesis. This means that both *MdMYB111* and *MdWRKY40* are important regulatory elements of the anthocyanin biosynthesis pathway (Figure 2) [36].

In addition to MYBs, other TFs are involved in regulating anthocyanin synthesis and transport. Anthocyanins are biosynthesized on the cytosolic surface of the endoplasmic reticulum and are then transported into the vacuole for storage [37]. Glutathione S-transferases (GSTs) are thought to be responsible for the transport of anthocyanins into the vacuole in apples [38]. *MdGSTF6* encodes an important GST transporter of anthocyanins in apple fruit and its expression is activated by *MdMYB1*, providing evidence for the related regulatory mechanisms [39]. Therefore, *MdMYB1* is thought to not only regulate anthocyanin synthesis, but also to control anthocyanin transport in apples [39]. A nitrate-induced LBD (lateral organ boundaries domain) TF gene, *MdLBD13*, has been discovered, which can repress anthocyanin biosynthesis by downregulating the expression of genes related to anthocyanin biosynthesis, such as *MdMYB1*, *MdMYB9/11*, and *MdbHLH3/33*, resulting in reduced anthocyanin accumulation [40]. *MdLBD13* could, thus, play the role of a negative regulator of anthocyanin biosynthesis [40].

In conclusion, MYB TFs, especially those encoded by *MdMYB1*, *MdMYBA*, and *MdMYB10* genes, play important roles in the regulation of apple skin color [14,27,28]. However, a number of other MYB family genes also control the anthocyanin biosynthesis pathway, such as the aforementioned *MdMYB3*, *MdMYB9*, *MdMYB11*, *MdMYB12*, *MdMYB16*, *MdMYB22*, *MdMYBDL1*, *MdMYB111*, and *MdMYB308*. Their effects in the anthocyanin pathway are shown in Figure 2.

### 3.2. Genetic Mapping

Since the 1990s, numerous quantitative genetics studies on apples have allowed the location of a huge number of quantitative trait loci (QTL) and major genes linked to the major agronomical traits. Amongst these, only a few were concerned with polyphenol compounds [41,42,43]. A small number of genetic studies have been performed to decipher red pigmentation in apple. Since apple skin color is one of the main factors defining the commercial value of apples, several genetic studies have been performed to decipher its locations in the genetic maps of apples. These studies have been performed thanks to the availability of different kinds of molecular markers, such as single sequence repeats (SSRs) [44]. However, the bulk of the studies used single nucleotide polymorphisms (SNPs) [41,45,46,47], which are the most abundant type of DNA sequence polymorphisms [48]. These were based on different origins and kinds of plant materials, such as segregating populations [41,45,46,49] and cultivar populations, through genome wide analysis studies (GWAS). The results of all these studies are convergent, showing that *MdMYB10* and *MdMYB1* are the two main transcription factors involved in the regulation of red skin color in apples [44]. As *MdMYB1* and *MdMYBA* share identical sequences and *MdMYB10* and *MdMYB1* genes are located at very similar positions, sharing 98% homology on linkage group 9, it was concluded that these genes were allelic [27,28,50,51]. In addition, MYB TFs also influence red-flesh apples, of which there are normally two types. In type 1, red pigmentation is observed from the fruit set through to maturity, exhibiting red fruit skin, leaves, stems, roots, and flowers. In type 2, red pigmentation is only present in the fruit cortex at the late stage of fruit development and the leaves are green [52,53]. Differences in anthocyanin accumulation in the red flesh type 2 apples have been associated with one SNP marker close to the *MdMYB10* homolog *MdMYB110a,* which is physically located on LG 17 [52].

The last genetic mapping results identified alleles of SSRs and SNPs markers that could be used in molecular assisted breeding [42,44,45,47]. However, these studies cannot assist in the distinction of apple mutants (e.g., Gala), which show very tiny differences in skin coloration. For this, new approaches need to be developed.

### 3.3. Copy Number Variations

Copy number variations (CNVs) are defined as deletions, duplications, or insertions of DNA sequence fragments longer than 50 base pairs in length [54]. CNVs have the potential to influence genes by altering their structure and expression [55]. CNVs are a common feature of plant genomes that include non-global duplication and deletion events [55].

Next-generation sequencing (NGS) technologies have greatly facilitated the discovery of CNVs [56]. Using NGS data, copy number variable regions (CNVRs) within the apple genome were detected, as well as examined for their distribution and impact [55]. In total, 876 CNVRs accounting for 3.5% of the apple genome were identified. The enrichment of the CNVRs with gene loci of agronomical significance has drawn attention to these newly discovered forms of genetic variation in apples [55].

Notably, some MYB TFs were found to have CNVs that could be linked to apple fruit coloration. For instance, a rearrangement in the upstream regulatory region of the gene encoding the *MdMYB10* was detected [57]. This rearrangement includes a series of multiple repeats, forming a microsatellite-like structure, featuring five direct tandem repeats of a 23 bp sequence. This *MdMYB10* rearrangement was only present in red-fleshed apple varieties and was not observed in white-fleshed varieties. Transient analysis showed that the 23 bp sequence motif is a target for the *MdMYB10* protein itself, and the number of repeat units is related to the increase in transactivation by *MdMYB10* [57]. The red-fleshed phenotype is also associated with enhanced expression of *MdMYB110a* (paralog of *MdMYB10*) [52]. Functional characterization of *MYB110a* showed that it could upregulate anthocyanin biosynthesis in tobacco (*Nicotiana tabacum L.*). *MdMYB10* (LG 9) and *MdMYB110a* (LG 17) have conserved functions in some varieties, but their expression and response to fruit maturity are different. *MdMYB10* was found to be expressed in apple skin, flesh, and foliage, while *MdMYB110a* was found to only be expressed in the fruit cortex [52]. Other anthocyanin-related MYBs were selected from a number of plant species, including apple, pear, strawberry, petunia, kiwifruit, and *Arabidopsis thaliana*, to initiate promoters containing the R6 motif, which has six minisatellite repeat units thought to increase anthocyanin pigmentation [58]. Insertion of the apple R6 motif into the *MYB10* orthologous promoter of pear (*PcMYB10*) and *Arabidopsis* (*AtMY75*) was able to increase anthocyanin levels [58].

In conclusion, copy number differences could be used as markers for apple color, especially *MdMYB10* and *MdMYB110a* (summarized in Table 1).

### 3.4. Transposable Elements

Transposable elements (TEs) are ubiquitous mobile genetic factors that can make up more than 50% of a plant’s nuclear genome [59]. They can be divided into two broad classes according to their method of proliferation [60,61]. Class I elements transpose by reverse transcription of RNA intermediates, and once inserted into a new location in the genome they cannot be removed [60,61]. This class includes retrotransposons and potential retroviruses with long terminal repeats (LTR), as well as non-LTR elements, including long and short interspersed nuclear elements (LINE and SINE, respectively) and processed pseudogenes [60,61]. Class II elements (also called DNA transposons) can be excised and inserted, and thus move from one nuclear position to another [60,61]. Some coding factors mediate their own transposition, while others are non-autonomous, depending on the activity of the transposase encoded at a separate site [60,61,62].

TEs are usually methylated via small RNAs, and this methylation can extend to surrounding genes, making them inactive [63,64]. The role played by retrotransposons in plant genomes is well known—by self-replication and insertion into multiple genomic sites [65,66], they have an influence on the size, structure, function, and evolution of plant genomes. Retrotransposons, thus, are an important source of genetic diversity; they may cause changes that could lead to genetic variations within plant species [66,67].

TEs account for 60% of the apple genome in the final assembly and provide insights into its evolution [68]. LTR retrotransposons (LRNs) are the most abundant type of TEs [69,70]. There is evidence that TEs affect the appearance of some genetic variation in the color of pome fruits [71]. Recently, a gypsy-like LTR retrotransposon (denoted as redTE) was found inserted in the upstream of the *MdMYB1* promoter and could be associated with red coloration [72]. The redTE controlled the development of red coloration by lowering the threshold of light response. This insertion appears in the HFTH1 (“Hanfu”, which has bright red skin color) genome and is located −3297 bp upstream of the ATG initiation codon of *MdMYB1* [72]. This redTE has two target site duplications (TSDs) and two identical flanking LTR sequences (1274 bp). Furthermore, redTE-mediated control of anthocyanin distribution patterns manipulates *MdMYB1* function through the creation of genetic and epigenetic alleles under natural conditions [72].

It is noteworthy that TEs were found to be associated with coloration in other fruit species. For example, in blood oranges, two different TE inserts are responsible for the cold-induced Ruby expression, thereby regulating fruit color [73]. In grapes, a Gypsy-like retrotransposon called Gret1 inhibited the expression of *MYBA1*, while the retrotransposon is linked with the development of white-skinned berries [74,75]. The recombination between Gret1 LTRs caused some restoration of MYB gene function and blush-skinned sports, including “Flame Muscat” and “Chardonnay Rose” [73,76]. Furthermore, it remains to be seen whether similar redTE insertions could enhance MYB transcription in other Rosaceae fruit species [72,73]. These findings strongly suggest that TE insertions may also play key roles in influencing traits of interest in apples.

In conclusion, redTE-mediated control of the distribution patterns of anthocyanin accumulation is exerted through the creation of genetic and epigenetic alleles that manipulate the function of *MdMYB1* under natural conditions. This suggests that some TEs and retrotransposon elements are linked with the anthocyanin biosynthesis pathway in apple skin. Therefore, we will next look at the epigenetic determinants of apple skin color.

## 4. Epigenetic Determinants of Apple Skin Color

In addition to genetic factors, some epigenetic factors can also affect apple skin color. Epigenetics describe mitotically or meiotically heritable variations in gene expression that cannot be explained by changes in DNA sequence [77,78,79]. Thus, epigenetics refers to the study of heritable changes in gene transcription that do not involve changes in the DNA sequence. Epigenetic marks can be transferred from one generation to the next [79,80]. Epigenetic regulation of gene expression is achieved through DNA methylation and specific histone modifications [79,81].

### 4.1. DNA Methylation and Demethylation

DNA methylation can be highly stable and inherited in a mendelian manner. This was demonstrated by experiments with 30 consecutive generations of plants in *Arabidopsis thaliana* [79,82]. In plants, DNA methylation has three sequence contexts: CG, CHG, and CHH (H = A, T, or C) [83]. One of the key activities of DNA methylation is the silencing of TFs to prevent their transcription and mobility.

Emerging evidence suggests that a modification in DNA methylation is also important for the ripening of fleshy fruits [84]. In tomatoes, the *VTE3* gene regulates the vitamin E content in fruits. *VTE3* expression is linked with the TE methylation level in the promoter region [85]. In sweet oranges, a DNA methylation inhibitor, by stopping further increase in DNA methylation, could prevent fruit from degreening [86]. Some studies have investigated the role of epigenetics in apples, comparing specific varieties and their mutants that produce fruit with a stable pigment pattern or different skin phenotypes, respectively. For example, mutants of “Honeycrisp” or “Fuji” can produce fruit with two different kinds of patterns,—striped and blushed. In “Honeycrisp”, DNA methylation at the *MdMYB10* promoter was detected’ increased DNA methylation levels were observed in green stripes, and a 900 bp long sequence, starting 1400 bp upstream of the translation start site, was highly methylated in *MdMYB10* [71]. Another example concerns varieties derived from “Gala”, with “Kidd’s D-8” (KID) being the red-skinned mutant and “Blondee” (BLO) being the yellow-skinned mutant [38]. Two regions, MR3 and MR7, exist in the *MdMYB10* promotor that showed significant differences between BLO and KID. Methylation was found to be higher and gradually increased during fruit development of BLO, whereas in KID it was lower and constant. The higher levels of DNA methylation reduced *MdMYB10* transcription, and thus anthocyanin production [38]. In “Hanfu”, DNA was also methylated in the MR3 and MR7 regions in *MdMYB1*; in the MR8-MR11 regions, the degree of methylation of redTE was high. This suggests that redTE-induced epigenetic changes may be related to variable color patterns [72].

DNA methylation could modify both regulatory and structural genes in the anthocyanin pathway. In apple skin, differential changes in methylation patterns related to anthocyanin concentrations have been reported [87]. Two differentially methylated regions (DMRs) and differentially expressed genes (DEGs) were linked to the anthocyanin pathway: *MdANS* and *MdF3H*. These genes were up regulated in apple mutants, and differences in the methylation patterns of their promoters were observed [87]. The transcription of structural genes may be regulated by both changes in TF levels and DNA methylation. Additionally, the expression of the TF *MdMYB114* was upregulated in deep-skinned apples [87]. In “Fuji”, the methylation in three ‘mutants with different colors was detected, and it was found that the CHH methylation level in the MR3 region (–1246 to –780) of the *MdMYB1* promoter was negatively correlated with *MdMYB1* expression [88]. *MdAGO4*, which plays a key role in RNA-directed DNA methylation, is required for CHH methylation, which was found to interact with the *MdMYB1* promoter. The promoter of *MdMYB1* was found to be methylated through binding of *MdAGO4* to this gene’s promoter, thereby regulating anthocyanin biosynthesis [88].

Methylation of cytosine in the genome of eukaryotes is often related to repeats, including TEs and their derivatives. Such sequences are usually enriched in centromeres and their vicinity [79]. It was confirmed that the differential accumulation of *MdMYB1*-specific mRNA was causing the difference in anthocyanin levels between “Granny Smith” and “Golden Delicious” [89]. The methylation level of the promoter region was linked to different levels of *MdMYB1* transcripts in the two varieties. The formation of red pigment in the skin of “Granny Smith” is related to the hypomethylation of the *MdMYB1* promoter [89].

Whole-genome bisulfite sequencing (WGBS) is a useful method of detection for methylation differences between different color mutants. Using this method, Li et al. [90] found the methylation relationship between Red Delicious (G0) and its four-generation bud sport mutants (G1 to G4) [90]. The phenotypes of the mutants were different and the pigmentation in the apple skin gradually increased from G0 to G4 [91]. The phenotype of these mutants in Red Delicious was obviously linked with the difference in DNA methylation [90]. Furthermore, in some flavonoid biosynthetic pathway genes (including CHS, PAL, F3’H, PER, 4CL, CYP98A, and CCoAOMT), the mCHG and mCG contexts are hypomethylated; the mCHG contexts upstream of *MdMYB10* especially cause the transcriptional activation and increase anthocyanin accumulation. However, the methylation of the mCG context upstream of *bHLH74* causes transcriptional repression and inhibits the accumulation of anthocyanin [90]. In summary, methylation is linked with apple skin coloration and patterning. Furthermore, higher levels of DNA methylation reduced *MdMYB10* expression, and thus anthocyanin production [88].

In addition to accumulation of DNA methylation having an influence on the anthocyanin pathway, demethylation is also linked to its regulation. Bagging treatment could induce epigenetic changes. Ma et al. [89] compared DNA methylation levels in the 2 kb upstream region of *MdMYB1* in bag-treated apples after removal of the bags and unbagged controls. There was a correlation between hypomethylation and the red-skinned phenotype in “Granny Smith” apples [89]. Granny Smith fruits responded to treatment with an inhibitor of DNA methylation, 5-aza-2′-deoxycytidine (5-aza-dC), an analog of cytosine that can inhibit the activity of DNA methyltransferases. These drug treatments effectively limited DNA methylation and induced color phenotype variants [92,93]. In Granny Smith, after bag removal and 5-aza-dC treatments, some *MdMYB1* promoter regions showed reduced DNA methylation. This could indicate a 5-aza-dC-treatment-induced activation of anthocyanin biosynthesis in Granny Smith skin [89]. Ma et al. [94] performed another study in the same year to compare the transcriptomes of Granny Smith skin with and without 5-aza-dC treatment after bagging [94]. They found many differentially expressed genes through 5-aza-dC-treated and non-treated apples, including TF genes and anthocyanin accumulation-related genes. Demethylation treatments were linked to differential gene expression, while the regulatory mechanisms were associated with red pigmentation in “Granny Smith” apples and other non-red apple fruits [94]. Demethylation also enhanced the expression of *MdMYB1-2* and *MdMYB1-3* and induced the accumulation of anthocyanin in “Mutsu”, which was obtained from a yellow-skinned cultivar [95]. Some cases of demethylation were documented for Fuji mutants. Usually, Fuji fruit exhibit a striped pattern; however, “Beni Shogun”, one of its mutants, has blushed skin. The difference in methylation between Fuji and Beni Shogun was linked to reduced DNA methylation at MR7 in the *MdMYB10* promoter, which increased full redness in the skin of these apples [96].

From this part, we can conclude that both gain and loss of DNA methylation can influence the anthocyanin pathway (Table 2). Furthermore, DNA methylation might inhibit anthocyanin biosynthesis in apple skin, while demethylation seems to induce it. However, epigenetic levels are different in different organizations and in different developmental stages in plants [97]. The methylation is inherited but is less stable which depending upon the environment [97]. For example, the chestnut methylation level decreases with the age of the tree [98]. Epialleles or epimutations can generate stable and heritable changes of fruit phenotypes [99]. However, in some situations epialleles could be less stable and the apple mutant skin color could revert to the original apple color; the reason causing this change is still unknown. These scientific questions still need to be researched in the future.

### 4.2. Histone Modifications

In recent years, there has been evidence suggesting that a conserved histone H2 variant, H2A.Z, and histone methylation are associated with the regulation of gene transcription in different organisms throughout the genome [100,101]. However, little is known about the roles of these two types of epigenetic regulation in the control of anthocyanin biosynthesis. The conserved histone H2 variant, H2A.Z, negatively controls the accumulation of anthocyanin in Arabidopsis [102]. H2A.Z and H3K4me3 have an antagonistic effect on the transcriptional regulation of anthocyanin biosynthesis genes, which highlights a role of chromatin in gene regulation and reflects the complexity of gene regulatory mechanisms [102].

Bagging treatment could induce epigenetic changes and the histone levels could be different in different regions of transcribed genes. This treatment was able to turn the normally non-red apple variety “Mutsu” into a red one. Furthermore, the histone H3K4me3 was found to be higher in the 5′ upstream region of *MdMYB1-2/-3*, while H3k27me3 was lower [95]. H3K4me3 and H3K27me3 in the 5′ upstream region of *MdMYB1-2/-3* were associated with the paper-bagging-induced red pigmentation. The modifications of the H3K4me3 and H3K27me3 could induce the expression of *MdMYB1-2/-3* in some regions [95].

An example for the link between histones and anthocyanin accumulation can be found in Arabidopsis. A HD-ZIP II TF HAT1 negatively regulates the accumulation of anthocyanin through post-translational regulation of the MYB-bHLH-WD40 (MBW) protein complex [103]. *MYB75* was found to form a transcriptional repressor complex with HAT1-TPL through the deacetylation of histone H3 at the target gene in Arabidopsis [103]. It was indicated that HAT1 inhibited the formation of MBW protein complexes and recruited TPL core inhibitors to epigenetically regulate the anthocyanin late biosynthesis genes (LBGs), thereby inhibiting the activity of MBW protein complexes, and thus anthocyanin accumulation [103]. In maize, the basic helix–loop–helix (bHLH) protein *R* interacts with the MYB TF *C1* and R-interacting factor 1 (RIF1) to form a C1-R-RIF1 complex [104]. This complex binds to the *A1* promoter and activates *A1* expression through increasing H3K9 and H3K14 acetylation levels in the promoter region [104].

In conclusion, identifying the interaction between MBW protein complexes and epigenetic regulators remains challenging [103]. How the histones regulate structural genes or TFs in the anthocyanins biosynthetic pathway in apple skin is still a complex question and an interesting subject that needs to be studied by subsequent researchers.

### 4.3. Small RNAs

Recently, in various organisms, small RNAs (sRNAs) have been identified as key genetic and epigenetic regulators, being involved in histone methylation and DNA modification to regulate the abundance of coding or non-coding RNAs [105]. In plants, microRNAs (miRNAs) and small interfering RNAs (siRNAs) are the main regulatory RNA species [105]. The former are mainly involved in post-transcriptional regulation, while the latter are linked with transcriptional regulation [105]. Many of these characteristic sRNAs are related to numerous biological programs, processes, and pathways in response to developmental cues, environmental stress, pathogen infection, and pest infestation [105].

In apples, miR828 and miR858 regulate a large number of MYB genes through targeting the region encoding the conserved R3 domain of the MYB protein [106]. MiR828 triggered the targeting of MYB genes to produce secondary phasiRNAs (phased siRNAs), thereby enhancing its silencing effect. Most MYBs for miR828 are linked with primary and secondary metabolism related to anthocyanin production [106]. Among them, there are nine MYBs co-targeted by miR828 and miR858, which are involved in the regulation of proanthocyanidin biosynthesis [106]. Xia et al. [106] conducted an analysis of 81 MYBs—29 MYBs were identified as targets, most of which are mainly involved in anthocyanin biosynthesis [106]. In tomato, miR858 plays a negative role in anthocyanin biosynthesis—blocking miR858 can increase the accumulation of anthocyanin by regulating the expression of *SlMYB7* and *SlMYB4861* [107].

The RdDM pathway could influence fruit coloration in different apple mutants. Jiang et al. [88] systematically studied the causes of coloring differences in different apple bud varieties and found that there was a significant negative correlation between the content of anthocyanin in the skin and the CHH methylation level of the TF *MdMYB1* promoter. CHH methylation is primarily mediated by the RdDM pathway [88]. The RdDM pathway AGO protein *MdAGO4-1/2* was found to interact with the apple *MdMYB1* promoter. In the apple callus, overexpression of *MdAGO4s* and *MdDRM2s* can increase the CHH methylation level at the *MdMYB1* promoter, thus affecting the accumulation of anthocyanins in apples [88]. In summary, apple *MdAGO4s* bind to ABS (AGO4 binding site) on the *MdMYB1* promoter, recruit the DNA methyltransferase *MdDRM2*, and exert a DNA methylation function. On the other hand, long non-coding RNAs (lncRNAs) mediate the methylation of *MdAGO4* binding site, thereby modifying the *MdMYB1* promoter, affecting its expression and regulating fruit color [88].

lncRNA could also be linked to anthocyanin biosynthesis in apples [108]. During light-induced rapid anthocyanin accumulation, RNA-seq analysis of apple skin of the “Red Fuji” variety revealed 5297 putative lncRNAs [108]. Differential expression analysis further showed that lncRNAs involved in photosynthesis during light treatment were transcribed [108]. It was predicted that two differentially expressed lncRNAs, *MLNC3.2* and *MLNC4.6*, are potential endogenous target mimics (eTMs) for miRNA156a, which can prevent cleavage of *SPL2-like* and *SPL33* during light-induced anthocyanin biosynthesis [108]. SPL (squamosa promoter-binding protein-like) interacts with MYB TFs and coordinates the biosynthesis of anthocyanins during the exposure of apple skin [108]. This research provides basic insights into the involvement of lncRNA in anthocyanin biosynthesis pathways of apple fruits [108].

In conclusion, small RNAs are now widely recognized as key genetic and epigenetic regulatory factors in various organisms and biosynthesis pathways, including that of anthocyanin biosynthesis. Small RNAs such as miR828 and miR858 seem to synergistically regulate a large number of MYB genes by directly targeting the region encoding the conserved R3 domain of the MYB protein.

## 5. Conclusions

In this review, we have summarized the recent research on the genetic and epigenetic mechanisms affecting the buildup of apple skin color. Most studies have investigated the genetic part of fruit coloring, with a focus on the genes in the flavonoid biosynthetic pathway, such as the *MdMYB1* gene, which is linked with apple skin color. However, evidence on the role of epigenetics in the development of fruit color and patterns is now mounting. A number of tasks remain to be performed in the future—this review has shown a surprisingly large variety of regulatory mechanisms involved in a host of different genetic and epigenetic factors underlying the expression of skin coloration in apple fruit. Although the number of studies dedicated to the topic is impressive, more work still needs to be performed to clarify the roles of certain factors, especially when mechanisms seem to be redundant or complementary. In addition, from a more practical point of view, breeding of certain apple varieties would benefit from the development of genetic and epigenetic markers. This would allow apple breeders to better and more reliably distinguish apple mutants, saving them time and money.

## Figures and Tables

**Table 1:** Overview of CNVs for apple color characteristics.

Related Gene	CNV Length	LG	Reference
*MdMYB10*	Five direct tandem repeats of a 23bp sequence	9	Espley et al. [28]
*MdMYB110a*	930-bp region upstream	17	Chagné et al. [52]

**Table 2:** Overview of methylation and demethylation in apple color characteristics.

Related Gene	Over (+) or under (−) Methylated	Effect on Transcription	Reference
*MdMYB10*	+	Reduces MdMYB10 transcription and anthocyanin production.	Telias et al. [71]; El-Sharkawy et al. [38]
*MdANS, MdF3H*	+	Modifies both regulatory and structural genes in the anthocyanin pathway.	Jiang et al. [87]
*MdMYB114*	+	Regulates anthocyanin biosynthesis in fully red apples	Jiang et al. [87]
*MdMYB1*	−	Formation of red pigment in the skin of Granny Smith.	Ma et al. [89]
*CHS, PAL, F3’H, PER,4CL, CYP98A, CCoAOMT*	−	Transcriptional activation and increase in anthocyanin accumulation.	Li et al. [90]
*MdbHLH74*	−	Transcriptional repression, inhibiting accumulation of anthocyanin.	Li et al. [90]

## Abbreviations

5-aza-dC	5-Aza-2′-deoxycytidine
ANS	Anthocyanidin synthase
CHI	Chalcone isomerase
CHS	Chalcone synthase
CNV	Copy number variation
CNVRs	Copy number variable regions
DEGs	Differentially expressed genes
DFR	Dihydroflavonol 4-reductase
DMG	Differentially methylated genes
DMR	Differentially methylated regions
DUS	Distinctness, uniformity, and stability
eTMs	Endogenous target mimics
F3H	Flavanone-3-hydroxylase
FLS	Flavonol synthase
GSTs	Glutathione *S*-transferases
LAR	Leucoanthocyanidin reductase
LDOX	Leucoanthocyanidin dioxygenase
LINE, SINE	Long and short interspersed nuclear elements
lncRNA	Long non-coding RNA
LRNs	LTR retrotransposons
LTR	Long terminal repeats
miRNAs	MicroRNAs
MRE	MYB recognition element
NGS	Next-generation sequencing
PA	Proanthocyanidin
phasiRNAs	Phased siRNAs
QTL	Quantitative trait locus
RdDM pathway	RNA-directed DNA methylation pathway
siRNAs	Small interfering RNAs
SNPs	Single Nucleotide Polymorphisms
sRNAs	Small RNAs
TE	Transposable elements
TF	Transcription factor
TSDs	Target site duplications
UFGT	UDP-glucose: flavonoid-3-*O*-glycosyltransferase
WGBS	Whole-genome bisulfite sequencing
